# Development and validation of an explainable machine learning model for predicting the risk of sleep disorders in older adults with multimorbidity: a cross-sectional study

**DOI:** 10.3389/fpubh.2025.1619406

**Published:** 2025-08-11

**Authors:** Xia Wang, Dan Zhang, Liu Lu, Shujie Meng, Yong Li, Rong Zhang, Jingjie Zhou, Qian Yu, Li Zeng, Jiang Zhao, Yu Zeng, Ru Gao

**Affiliations:** ^1^School of Basic Medical Sciences and School of Nursing, Chengdu University, Chengdu, China; ^2^Rehabilitation Department, Sichuan Provincial People’s Hospital East Sichuan Hospital and Dazhou First People’s Hospital, Dazhou, China; ^3^Nursing Department, The Fourth People’s Hospital of Yibin, Yibin, China; ^4^Rehabilitation College, Sichuan Health Rehabilitation Vocational College, Zigong, China; ^5^Tellyes Scientific Inc., Tianjin, China; ^6^Nursing Department, The People’s Hospital of Wenjiang Chengdu, Chengdu, China

**Keywords:** machine learning, multimorbidity, older adults, sleep disorder, prediction model

## Abstract

**Objective:**

To develop and validate an explainable machine learning model for predicting the risk of sleep disorders in older adults with multimorbidity.

**Methods:**

A total of 471 older adults with multimorbidity were recruited between October and November 2024. We employed six machine learning (ML) methods, namely logistic regression (LR), neural network (NN), support vector machine (SVM), gradient boosting machine (GBM), K-Nearest Neighbors (KNN), and light gradient boosting machine (LightGBM), to predict the risk of sleep disorders based on their sociodemographic data, health behavior factors, mental health, and disease-related data. The optimal model was identified through the evaluation of the area under the curve (AUC). This study also employed explainable machine learning techniques to provide insights into the model’s predictions and outcomes using the SHAP (Shapley Additive Explanations) approach.

**Results:**

The prevalence of sleep disorders was 28.7%. Among the six models developed, the GBM model achieved the best performance with an AUC of 0.881. The analysis of feature importance revealed that the top seven predictors of sleep disorders were frailty, cognitive status, nutritional status, living alone, depression, smoking status, and anxiety.

**Conclusion:**

This study is the first to predict sleep disorders in Chinese older adults with multimorbidity using explainable machine learning methods and to identify seven significant risk factors. The SHAP method enhances the interpretability of machine learning models and helps medical staff better understand the rationale behind the predicted outcomes more effectively.

## Introduction

The global population is aging rapidly, and it is estimated that the number of older adults will reach around 1.5 billion by 2050 (1). However, the issue of population aging is particularly severe in China. As shown by the seventh national population census, the number of people aged 60 and over has exceeded 264 million, representing 18.70% of China’s total population (2). With the growing aging population and increasing life expectancy, there has been a substantial rise in the prevalence of chronic diseases. This increase has adversely affected the quality of life and compromised the physical and mental health of individuals (3). The World Health Organization (WHO) defines multimorbidity as the co-occurrence of two or more chronic conditions within an individual (4). The mechanisms underlying multimorbidity are complex and are influenced by multiple factors. Additionally, different chronic diseases that co-occur may share the same risk factors (5). Older adults are more prone to multimorbidity due to weakened physical functions and reduced immunity (6). Previous studies have demonstrated a higher prevalence of multimorbidity in older adults compared with the general population, and this proportion increases further with age (7, 8). This is a huge challenge for patients, medical staff and even the entire medical and health service system.

Sleep disorders, including insomnia, hypersomnia, circadian rhythm disturbances, sleep-related breathing issues, narcolepsy, and parasomnias, are particularly common in older adults with multimorbidity (9). Furthermore, several studies have found that sleep disorders can impair neurocognitive function, the motor system, and the immune system, consequently increasing the risk of falls, hospital admissions, and mortality (10, 11). The prevalence of sleep problems among the in older adults with multimorbidity has always been high. It has been reported that the prevalence of sleep disorders among older adults with multimorbidity is approximately 59% (12). A cross-sectional study utilizing data from over 200,000 individuals found that the incidence of lifetime insomnia among those with two or more health conditions, including hypertension, diabetes, stroke, heart disease, cancer, hip fracture and other fractures, was 2.6 times higher than in the healthy population (13). Accordingly, identifying the predictors of sleep disorders in older adults with multimorbidity is essential for timely interventions and preventing adverse clinical outcomes.

Numerous studies have explored risk factors for sleep disorders in healthy older adults (14, 15). Furthermore, some studies have indicated significant differences in both the clinical characteristics and prevalence of sleep disorders between healthy older adults and those with multimorbidity (16, 17). However, few studies have focused on identifying the risk factors for sleep disorders in older adult people with multimorbidity. Therefore, developing predictive models for older adult individuals with multimorbidity is an upstream approach to preventing sleep disorders.

To date, sleep disorder prediction models have been developed for older adults with conditions such as stroke (18, 19), coronary heart disease (20), chronic kidney disease (CKD) (21, 22), hypertension (23), cancer (24, 25), asthma (26) and chronic obstructive pulmonary disease (COPD) (27). However, most prediction models focus on a single chronic disease. In contrast, there is currently no predictive model for sleep disorders that has been specifically developed for older adult patients with multimorbidity. In addition, most risk models for sleep disorders are based on traditional logistic regression methods. Logistic regression models (28, 29), constrained by rigid linearity assumptions and limited capacity to automatically capture interaction effects between variables, exhibit heightened sensitivity to multicollinearity, high-dimensional data, and sample size variations. These limitations often result in biased coefficient estimation, overfitting, and compromised generalization performance, thereby restricting their applicability in complex medical prediction scenarios. Yang et al. (30) developed a sleep disorder prediction model using logistic regression, which achieved a relatively low AUC of 0.678 (95% CI: 0.635–0.720), along with suboptimal sensitivity (69.4%) and specificity (59.6%). Moreover, Armon et al. (31) conducted logistic regression to predict the incidence of insomnia at an 18-month follow-up. Their model, which controlled for confounders such as depression and neuroticism, yielded an odds ratio for the predictive effect of baseline burnout on subsequent insomnia. However, the study also highlighted the challenges posed by multicollinearity among predictors, including age, body mass index, and depressive symptomatology, which could potentially distort the estimation of regression coefficients and limit the model’s predictive accuracy. These findings collectively underscore the inherent limitations of logistic regression in handling complex interactions and high-dimensional data, thereby compromising the robustness and generalizability of sleep disorder prediction models.

In recent years, many studies have begun to utilize machine learning (ML) models for predicting various diseases or clinical conditions, achieving superior performance compared with traditional statistical methods (32–34). Machine learning (ML) models can capture intricate, non-linear relationships and previously unknown correlations within data, thereby providing deeper insights into clinical datasets (35). Consequently, ML models offer significant potential for use in clinical settings where large volumes of data are processed and the relationships between clinical characteristics and outcomes remain unclear (36). A study (37) conducted in Qatar utilized physical activity data derived from sleep time to apply various ML methods, including multilayer perceptron (MLP), convolutional neural network (CNN), simple Elman-type recurrent neural network (RNN), long short-term memory recurrent neural network (LSTM-RNN), and a time-batched version of LSTM-RNN (TB-LSTM), to predict insomnia in the older adult and compared these methods with traditional logistic regression. ML models outperform traditional logistic regression, as they can address the limitations of statistical methods and develop personalized risk predictions. Although ML methods have great advantages, their practical clinical applications remain limited by many factors. The performance of numerous predictive models has not been assessed with respect to discrimination, calibration, clinical utility. Moreover, the interpretability of their prediction results is limited, which restricts their general applicability and operability. Consequently, this study aimed to develop and validate an ML model for predicting sleep disorders in older adults with multimorbidity. The model also utilized the Shapley Additive Explanations (SHAP) (38) approach to interpret the results, thereby enabling targeted interventions to modify risk factors and support clinical decision-making.

## Methods

### Study design

In this cross-sectional study, we utilized multi-stage stratified cluster random sampling to recruit participants from communities in Yibin City, Sichuan Province, China, between October and November 2024. Data were collected via in-person interviews with participants, based on expert opinions and an extensive literature review. The data included general information, health behaviors, social support, anxiety, depression, sleep conditions, cognitive status, frailty, nutritional status, activity status, and chronic disease conditions. We then employed six ML methods to construct models for identifying the risk of sleep disorders and compared their performance to determine the optimal model. Furthermore, the SHAP approach was used to interpret the results of the best-performing model. This study adhered to the transparent reporting of a multivariable prediction model for individual prognosis or diagnosis + AI (TRIPOD+AI) guidelines (39) for prediction model development, validation, and performance evaluation.

### Participants

In this study, 471 older adults with multimorbidity were recruited from communities. Inclusion criteria: (a) age ≥ 60 years; (b) meeting the World Health Organization (WHO) diagnostic criteria for multimorbidity, defined as the co-occurrence of two or more chronic conditions in an individual, including any of the following 14 chronic diseases: hypertension, dyslipidaemia, chronic lung disease, stroke, diabetes or hyperglycaemia, heart disease, cancer or malignant tumors, liver disease, kidney disease, gastric disease or other digestive disorders, arthritis, rheumatism, or asthma; (c) having language communication skills. Exclusion criteria: (a) mental illness or memory-related diseases; (b) the presence of other serious diseases that hinder participation in the survey.

### Sample size

Following the application of inclusion and exclusion criteria, 471 older adults were deemed eligible for participation. The dataset was then randomly divided into a training set and a validation set at a ratio of 7:3. The calculation principle for sample size in the ML algorithm is that the number of events per variable (EPV) should be ≥10. The minimum sample size required for modeling is 252. In our study, there are 330 participants in the training set, which meets the sample size requirements.

### Outcome

Sleep disorders were measured using the Pittsburgh Sleep Quality Index (PSQI) (40), which is a self-administered scale comprising seven components related to sleep over the past month. These components include subjective sleep quality, sleep latency, sleep duration, habitual sleep efficiency, sleep disturbances, use of sleep medication, and daytime dysfunction. Each component is scored on a 4-point Likert scale, ranging from 0 to 3. The total score ranges from 0 to 21, with higher scores indicating poorer sleep quality. A total score of ≥ 5 denotes poor sleep quality (41, 42). Additionally, this study converted the PSQI scores into binary variables and employed them as outcome variables.

### Potential predictors

#### Demographics

Sociodemographic variables included gender, age, marital status, education level, source income, body mass index (BMI), drinking status, smoking status, and frequency of hospital visits.

#### Frailty

The instruments used to assess frailty is the FRAIL (Fatigue, Resistance, Ambulation, Illnesses, and Loss of Weight) scale (43, 44). The scale has the advantages of being simple, effective and widely applicable. Furthermore, it can comprehensively assess the physical function, cognitive ability, emotional state and social activities of the older adult. The scale comprises five items, each scored on a 5-point scale ranging from 0 to 5. The total score ranges from 0 to 25, with 0 indicating no frailty, 1–2 indicating pre-frailty, and 3 or above indicating frailty.

#### Cognition state

The Mini-Mental State Examination (MMSE) (45) was utilized to assess cognitive function in older adults and ranks as one of the predominant cognitive screening tools employed in clinical practice. This scale can quickly identify whether a patient has cognitive dysfunction and help doctors make an early diagnosis and intervention. The scale comprises 30 questions and covers five dimensions: orientation, memory, attention and calculation, recall, and language ability. Scores range from 0 to 30, with 0–26 indicating cognitive impairment and 27–30 indicating normal cognitive function.

#### Depression

The Geriatric Depression Scale-15 (GDS-15) (46) is a self-rated scale that was used to detect depression and assess the severity of depressive symptoms in older adults in the past week. The scale contains 15 items, with each item scored at either 0 or 1 point. The total score ranges from 0 to 15, with a score exceeding 5 points indicating the presence of depressive symptoms.

#### Social support

Social support was assessed using the Social Support Rating Scale (SSRS) (47), a tool developed by Xiao. This scale consists of 10 items and is structured around three dimensions: objective support, subjective support, and the utilization of support. The total score ranges from 0 to 66, with 0–22 corresponding to low social support, 23–44 to moderate social support, and 45–66 to high social support.

#### Nutritional status

Nutritional status was assessed using the Mini Nutritional Assessment (MNA) (48), which is a widely used tool in clinical practice for assessing malnutrition in older adults and mainly includes four parts: anthropometric measurement, comprehensive assessment, dietary status, and subjective evaluation. It contains 18 items, with a total score ranging from 0 to 30 points. A score of ≥24 indicates normal nutritional status, a score of 17–23.5 indicates a risk of malnutrition, and a score of <17 indicates malnutrition.

#### Capability of daily living activities

Daily living activities were assessed using the Activities of Daily Living (ADL) Scale (49), developed by Lawton and Brody in the United States in 1969. This scale primarily evaluates participants’ functioning in everyday life, comprising 14 items: six from the Physical Self-Maintenance Scale and eight from the Instrumental Activities of Daily Living Scale. Total scores range from 14 to 56, with higher scores indicating greater impairment in daily living activities. A total score of 22 or above signifies a functional limitation.

### Data preprocessing and feature selection

Data preprocessing was primarily used to enhance data quality and improve model performance. We addressed missing values through deletion and estimation. First, participants with over 20% missing values were excluded, after which the missing data were imputed using multiple imputation techniques. Multiple imputation (50) is considered a statistical technique for replacing missing data while accounting for the uncertainty inherent in missing values, thereby reducing bias and improving the accuracy of the analysis.

### Feature selection

To enhance the predictive performance of the model in this study, feature selection was conducted using the Least Absolute Shrinkage and Selection Operator (LASSO) (51) and the Boruta (52). LASSO can solve the problems of high dimensionality and multicollinearity between variables. However, while LASSO can effectively mitigate overfitting and select features by penalizing coefficient magnitudes, its efficacy is constrained by the penalty parameter’s strength and it presumes a linear relationship, thus failing to capture interactions and complex patterns within the data (53). The optimal parameter (*λ*) in the LASSO model was selected via 10-fold cross-validation. The LASSO regularization path identified lambda.min as the optimal λ value, corresponding to the predictor variables with non-zero coefficients. Boruta, a feature selection method founded on Random Forest, addresses these limitations by identifying important features through comparison with their shuffled counterparts (54). The Boruta algorithm selects variables by comparing the importance scores of original variables with those of randomly generated shadow features, iteratively retaining variables that significantly outperform shadow features. In our study, LASSO and Boruta were used to screen variables separately, and their intersection was taken as the final set of predictive variables. Only predictors identified by both LASSO and Boruta were included to ensure consistency across methods.

### ML models

Given the imbalance between positive and negative events in the dataset, the Synthetic Minority Over-sampling Technique (SMOTE) (55) was employed to address this issue. SMOTE is a widely used oversampling technique for addressing imbalanced datasets. It generates additional minority class samples by leveraging the k-nearest neighbors of each minority instance, helping to balance the distribution between the minority and majority classes. The dataset was randomly allocated to a training set (70%) for model development and an internal validation set (30%) for model assessment. To prevent model overfitting, 10-fold cross-validation was implemented. We utilized the following six representative ML algorithms to construct the predictive models: logistic regression (LR), support vector machine (SVM), gradient boosting machine (GBM), neural network (NN), K-Nearest Neighbors (KNN), and LightGBM.

### Model performance and evaluation

The validation set was utilized to evaluate model performance. Internal validation performance was assessed by computing the means and 95% confidence intervals (CIs) of the area under the receiver operating characteristic curve (AUROC). Furthermore, the ML model with the optimal performance was selected based on its AUC value. The AUC of the different models was compared using the DeLong test (56). The models were also evaluated using accuracy, sensitivity, and specificity. A calibration curve was constructed to examine the consistency between predicted probabilities and actual outcomes. Decision curve analysis (DCA) was performed to evaluate the practical utility of the model in clinical decision-making and to calculate the net benefit.

### Model interpretation

The interpretability of the function modeled by ML is only partially limited by the “black-box” nature of these algorithms. To enhance interpretability, we applied the Shapley Additive Explanations (SHAP) (55) approach to evaluate the significance of features within the model. SHAP, rooted in game theory, is a widely used approach for explaining the outputs of ML models. Its fundamental principle involves quantifying the contribution of each individual feature by evaluating its influence on the cooperative prediction process. In SHAP, each feature is assigned an importance value, known as the SHAP value, which ensures a fair distribution of predictive influence across different variables. A higher mean absolute SHAP value denotes greater feature importance in predicting sleep disorders. A positive SHAP value indicates that the corresponding feature is associated with a higher risk of sleep disorders, while a negative SHAP value suggests that the feature is linked to a lower risk. Conversely, a SHAP value close to zero indicates little to no association between the feature and the prediction. The SHAP model offers several advantages. First, it provides global interpretability by quantifying the contribution of each feature to the target outcome—whether positive or negative. Second, it delivers local interpretability, as each individual prediction is assigned its own set of SHAP values.

### Statistical analysis

Statistical analyses were conducted using R 4.3.2 and SPSS 25.0. Continuous variables were summarized as mean ± standard deviation (SD), while categorical variables were presented as frequency (percentage). Group comparisons were performed using the chi-square test. Finally, we further validated the relationship between machine learning-selected features and sleep disorders using logistic regression. First, we constructed univariate regression models, then included statistically significant variables from these analyses in a multivariate model to examine the independent effects of each variable. A two-sided *p*-value < 0.05 was considered statistically significant.

## Results

### Characteristics

This study included 471 older adults with multimorbidity, of whom 131 (28.7%) experienced sleep disorders. The study population consisted of 216 males (45.9%) and 255 females (54.1%), with a mean age of 74.08 ± 6.82 years. Regarding marital status, 289 participants (61.4%) had a spouse, while 182 (38.6%) were unmarried or widowed. Patient characteristics are detailed in Table 1. Significant differences were observed between the sleep disorder group and the non-sleep disorder group in terms of marital status, living alone, frequency of hospital visits, smoking status, BMI, social support, depression, anxiety, cognitive status, frailty, and nutritional status (*p* < 0.05).

**Table 1:** Baseline characteristics of the participants with or without sleep disorders.

Variables	Total (*n* = 471)	Non-sleep disorders (*n* = 340)	Sleep disorders (*n* = 131)	*χ* ^2^	*P*
Gender (%)				0.086	0.769
Female	216 (45.9)	154 (45.3)	62 (47.3)		
Male	255 (54.1)	186 (54.7)	69 (52.7)		
Age (years) (%)				2.441	0.295
60–69	142 (30.1)	109 (32.1)	33 (25.2)		
70–79	232 (49.3)	165 (48.5)	67 (51.1)		
≥80	97 (20.6)	66 (19.4)	31 (23.7)		
Marital status (%)				5.280	0.022
Spouse	289 (61.4)	220 (64.7)	69 (52.7)		
No spouse	182 (38.6)	120 (35.3)	62 (47.3)		
Education level (%)				1.950	0.583
Primary or below	305 (64.7)	216 (63.5)	89 (67.9)		
Lower secondary	105 (22.3)	78 (22.9)	27 (20.6)		
Upper secondary	47 (10.0)	37 (10.9)	10 (7.6)		
College or above	14 (3.0)	9 (2.7)	5 (3.9)		
Living alone (%)				18.148	<0.001
No	411 (87.3)	311 (91.5)	100 (76.3)		
Yes	60 (12.7)	29 (8.5)	31 (23.7)		
Source income (%)				5.472	0.140
Family	179 (38.0)	122 (35.9)	57 (43.5)		
Pensions	178 (37.8)	128 (37.6)	50 (38.2)		
Government subsidies	79 (16.8)	65 (19.1)	14 (10.7)		
Others	35 (7.4)	25 (7.4)	10 (7.6)		
Frequency of hospital visits (year) (%)				9.431	0.009
0	123 (26.1)	100 (29.4)	23 (17.6)		
1–2	287 (60.9)	203 (59.7)	84 (64.1)		
≥3	61 (13.0)	37 (10.9)	24 (18.3)		
Smoking (%)				6.801	0.009
No	370 (78.6)	278 (81.8)	92 (70.2)		
Yes	101 (21.4)	62 (18.2)	39 (29.8)		
Drinking (%)				0.187	0.666
No	468 (99.4)	337 (99.1)	131 (100)		
Yes	3 (0.6)	3 (0.9)	0 (0)		
BMI (%)				14.507	0.002
<18.5	41 (8.7)	20 (5.9)	21 (16)		
18.5–23.9	229 (48.6)	175 (51.5)	54 (41.2)		
24–27.9	141 (29.9)	105 (30.9)	36 (27.5)		
≥28	60 (12.8)	40 (11.7)	20 (15.3)		
Social support (%)				11.917	0.003
Low	198 (42.1)	128 (37.6)	70 (53.4)		
Medium	207 (43.9)	156 (45.9)	51 (38.9)		
High	66 (14.0)	56 (16.5)	10 (7.7)		
Depression (%)				14.980	<0.001
No	402 (85.4)	304 (89.4)	98 (74.8)		
Yes	69 (14.6)	36 (10.6)	33 (25.2)		
Anxiety (%)				7.379	0.007
No	459 (97.5)	336 (98.8)	123 (93.9)		
Yes	12 (2.5)	4 (1.2)	8 (6.1)		
Cognitive impairment (%)				19.334	<0.001
No	215 (45.6)	177 (52.1)	38 (29)		
Yes	256 (54.4)	163 (47.9)	93 (71)		
Frailty (%)				21.937	<0.001
No	227 (48.2)	180 (52.9)	47 (35.9)		
Pro	188 (39.9)	133 (39.1)	55 (42)		
Yes	56 (11.9)	27 (8.0)	29 (22.1)		
Nutritional status (%)				23.150	<0.001
Good	176 (37.4)	141 (41.5)	35 (26.7)		
Risk	255 (54.1)	182 (53.5)	73 (55.7)		
Bad	40 (8.5)	17 (5)	23 (17.6)		
Activity limitation (%)				0.003	0.960
No	440 (93.4)	317 (93.2)	123 (93.9)		
Yes	31 (6.6)	23 (6.8)	8 (6.1)		

BMI, body mass index.

### Preprocessing of data and screening of variables

**Figure 1:**
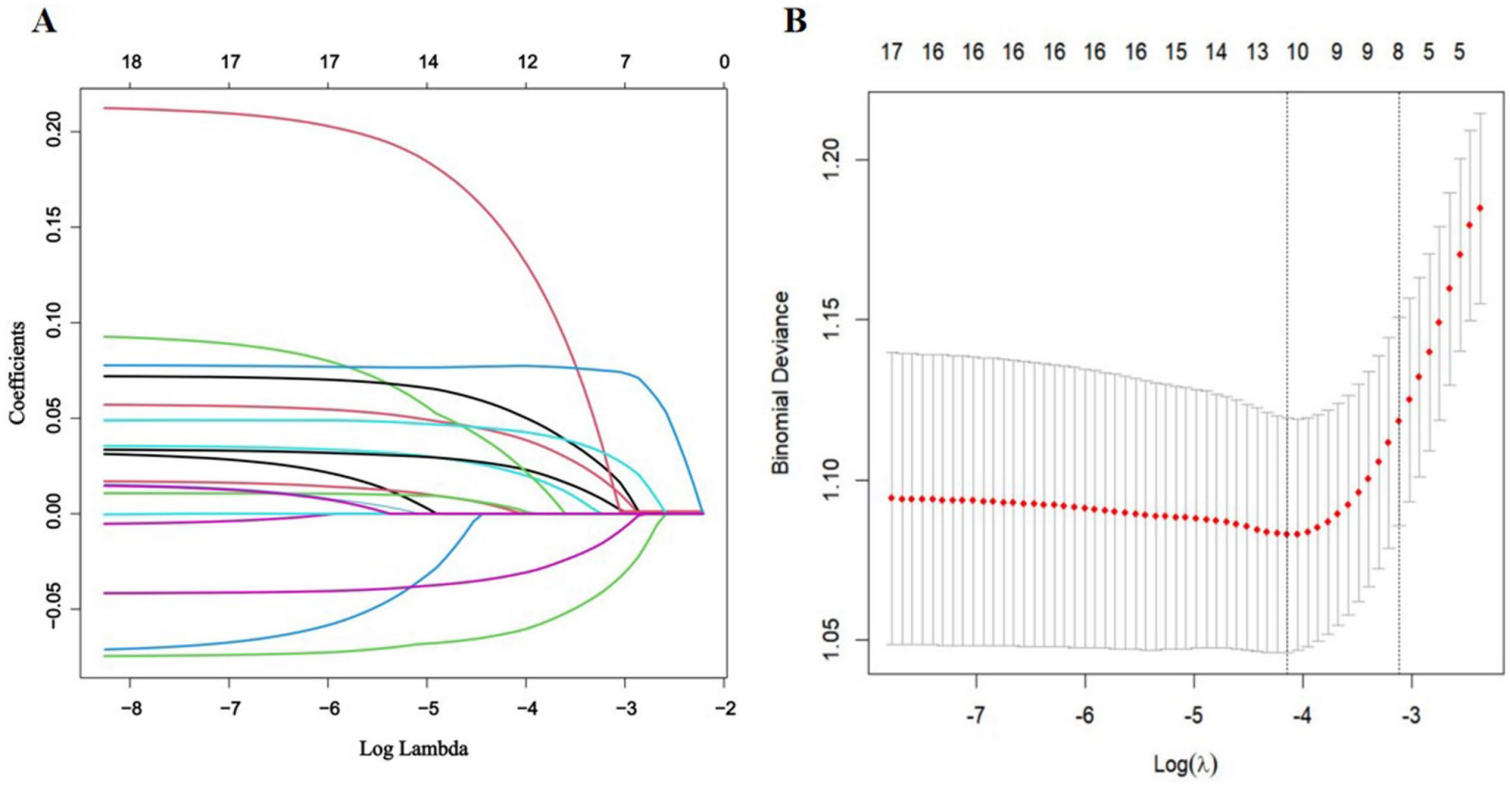
Feature selection via LASSO regression. **(A)** The coefficient profile plot was produced versus the log (*λ*). **(B)** The adjustment parameter (λ) was screened using 10-fold cross-validation in the LASSO model. The binomial deviance curve was plotted against log (λ). The dotted vertical lines indicated the optimal predictors using the minimum criteria (min. criteria) and the 1 standard error (SE) of the minimum criteria (1-SE criteria).

**Figure 2:**
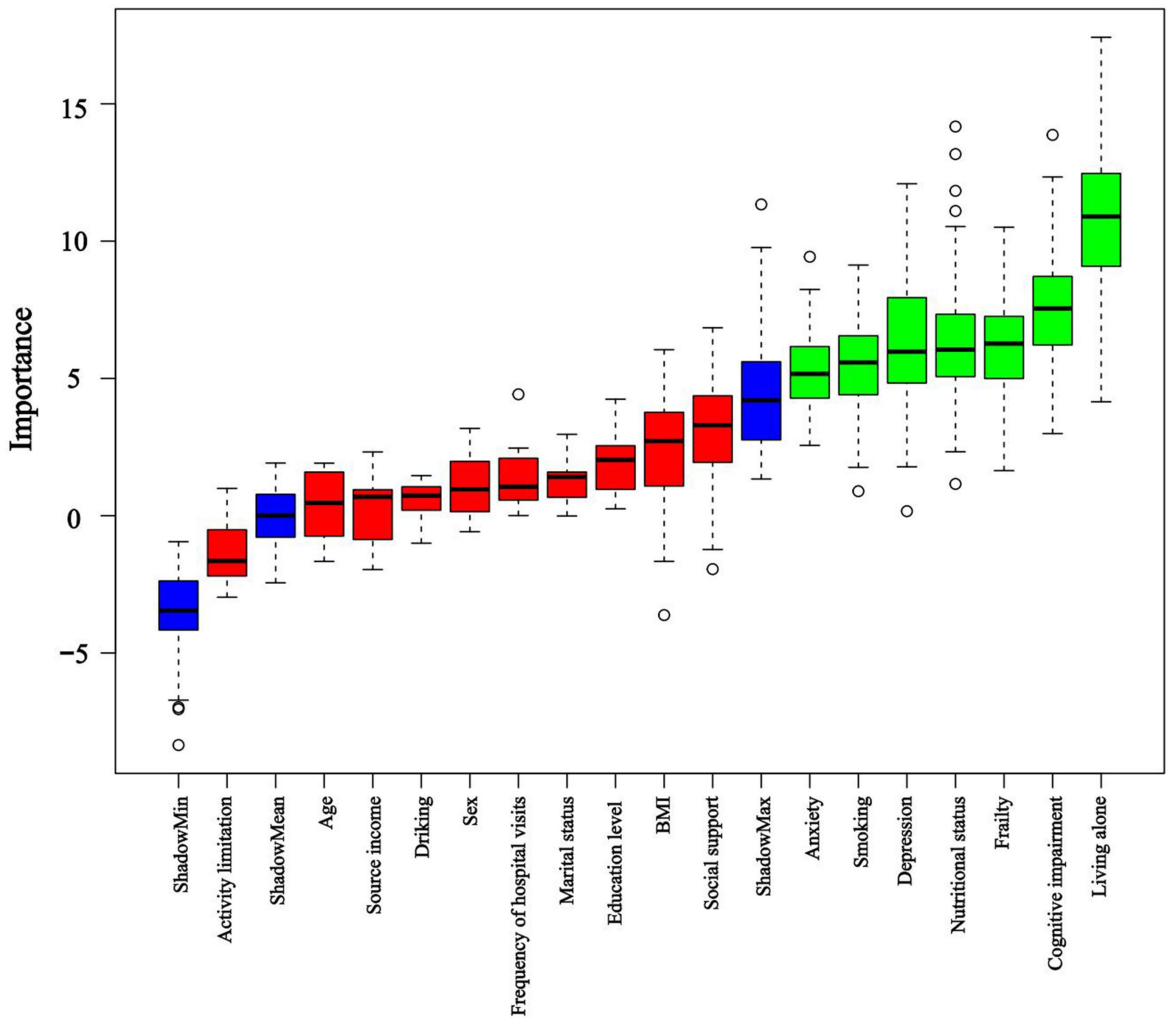
Importance of shadow and predictor variables selected by the Boruta algorithm. Blue boxplots correspond to the minimum, average, and maximum Z scores of a shadow attribute. The Z-score clearly separates important and non-important attributes. Red and green colors represent rejected and confirmed attributes selected by Boruta, respectively.

LASSO and Boruta were used to select relevant variables from the included indicators. As shown in Figure 1, the optimal parameter (*λ*) selection in the LASSO model was determined using 10-fold cross-validation. The LASSO regularization path selected lambda.1 min as the predictor variable with 10 non-zero coefficients corresponding to the optimal Log (λ) value, including living alone, frequency of hospital visits, smoking, BMI, social support, depression, anxiety, cognitive status, frailty, and nutritional status. As shown in Figure 2, the Boruta algorithm identified seven important features: living alone, smoking status, depression, anxiety, cognitive status, frailty, and nutritional status. The final set of predictors was determined by the intersection of the two methods, resulting in the selection of living alone, smoking, depression, anxiety, cognitive status, frailty, and nutritional status. To address class imbalance, the SMOTE was applied to resample the training set. After SMOTE-based oversampling, the sample sizes of the sleep disorders and non-sleep disorders groups reached 184 each, achieving data balance (Supplementary Table S1).

### Model performance and comparison

**Figure 3:**
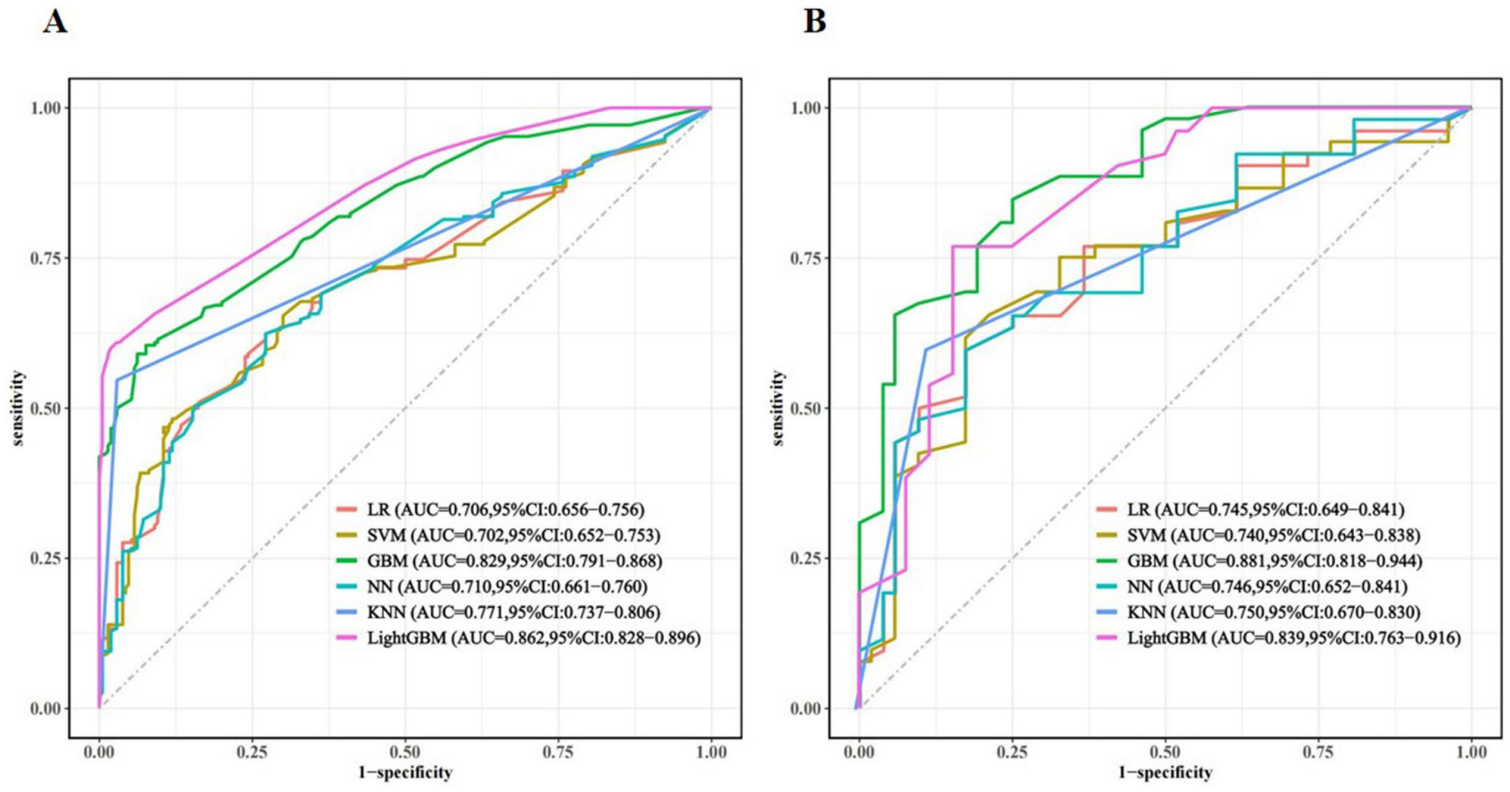
Receiver-operating characteristic curve (ROC) for different machine learning models in the training set **(A)** and validation set **(B)**.

Seven variables, including living alone, smoking status, depression, anxiety, cognitive status, frailty, and nutritional status, were selected as predictors. The dataset was partitioned randomly into training set (*n* = 330) and validation set (*n* = 141) at a 7:3 ratio, as detailed in Table 2. Prediction models were constructed using six ML algorithms: LR, SVM, GBM, NN, KNN, and LightGBM. Model performance was evaluated using 10-fold cross-validation, and the detailed results are presented in Table 3. The AUC values ranked the models in the validation set from highest to lowest as follows: GBM, LightGBM, KNN, NN, LR, and SVM. The GBM exhibited superior performance, achieving an AUC of 0.881, accuracy of 0.798, sensitivity of 0.864, specificity of 0.750, precision of 0.772, and an F1-score of 0.807. The ROC curves for the training and validation sets of each model are presented in Figure 3. The AUC value of the GBM model’s ROC curve was higher than those of the other five models, and significant differences (*p* < 0.05) were found among other models (Supplementary Table S2, DeLong test). Subgroup analyses were performed by sex and age. Sex-stratified validation demonstrated comparable generalization performance between males and females, with both groups achieving optimal AUC values using either GBM or LightGBM models (Supplementary Figure S1). Age-stratified analysis revealed superior generalization in the ≥80 years subgroup, with all three age groups attaining high AUC performance through GBM or LightGBM models (Supplementary Figure S2).

**Table 2:** Comparison of characteristics between training set and validation set.

Variables	Training set (*n* = 330)	Validation set (*n* = 141)	*P*
Sleep disorders (%)			0.542
No	238 (72.1)	102 (72.3)	
Yes	92 (27.9)	39 (27.7)	
Living alone (%)			0.643
No	289 (87.6)	122 (86.5)	
Yes	41 (12.4)	19 (13.5)	
Frequency of hospital visits (year) (%)			0.814
0	85 (25.8)	38 (27.0)	
1–2	204 (61.8)	83 (58.8)	
≥3	41 (12.4)	20 (14.2)	
Smoking (%)			0.948
No	258 (78.2)	112 (79.4)	
Yes	72 (21.8)	29 (20.6)	
Depression (%)			0.142
No	275 (83.3)	127 (90.1)	
Yes	55 (16.7)	14 (9.9)	
Anxiety (%)			1.000
No	320 (97.0)	139 (98.6)	
Yes	10 (3.0)	2 (1.4)	
Cognitive impairment (%)			1.000
No	147 (44.5)	68 (48.2)	
Yes	183 (55.5)	73 (51.8)	
Frailty (%)			0.608
No	154 (46.7)	73 (51.8)	
Pro	129 (39.1)	59 (41.8)	
Yes	47 (14.2)	9 (6.4)	
Nutritional status (%)			0.446
Good	124 (37.6)	52 (36.9)	
Risk	176 (53.3)	79 (56.0)	
Bad	30 (9.1)	10 (7.1)	

**Table 3:** The performance comparison of six machine learning models in validation set.

Model	AUC (95%CI)	Accuracy	Sensitivity	Specificity	Precision	F1
GBM	0.881 (0.818–0.944)	0.798	0.846	0.750	0.772	0.807
LightGBM	0.839 (0.763–0.916)	0.808	0.769	0.846	0.833	0.800
KNN	0.750 (0.670–0.830)	0.750	0.615	0.885	0.842	0.711
NN	0.746 (0.652–0.841)	0.712	0.596	0.827	0.775	0.674
LR	0.745 (0.648–0.841)	0.712	0.596	0.827	0.775	0.674
SVM	0.740 (0.643–0.838)	0.721	0.654	0.788	0.756	0.701

GBM, gradient boosting machine; LghtGBM, light gradient boosting machine; KNN, k-Nearest Neighbors; NN, neural network; LR, logistic regression; SVM, support vector machine; AUC, the area under the curve.

**Figure 4:**
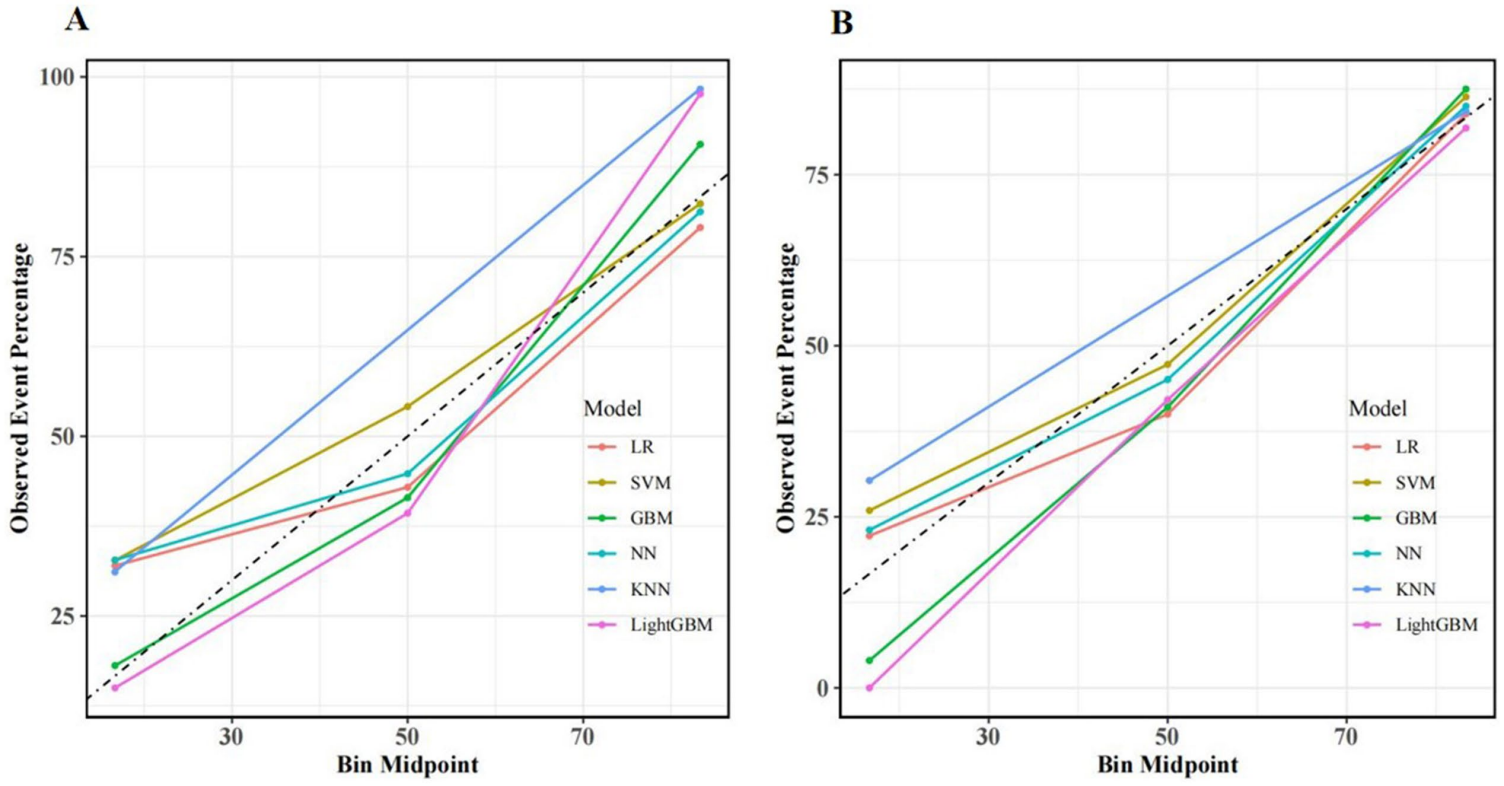
Calibration curves for different machine learning models in the training set **(A)** and validation set **(B)**.

The model’s calibration was assessed through the use of calibration curves, which evaluate the concordance between actual and predicted probabilities. If the calibration curve is close to the diagonal, this indicates good agreement between the predicted and observed probabilities. In our study, internal validation of the model demonstrated that the calibration curves of several ML algorithms, including GBM, LightGBM, NN, LR, and SVM, showed good calibration performance. However, the performance of KNN was slightly inferior. Calibration curves for the training and validation sets are shown in Figure 4.

**Figure 5:**
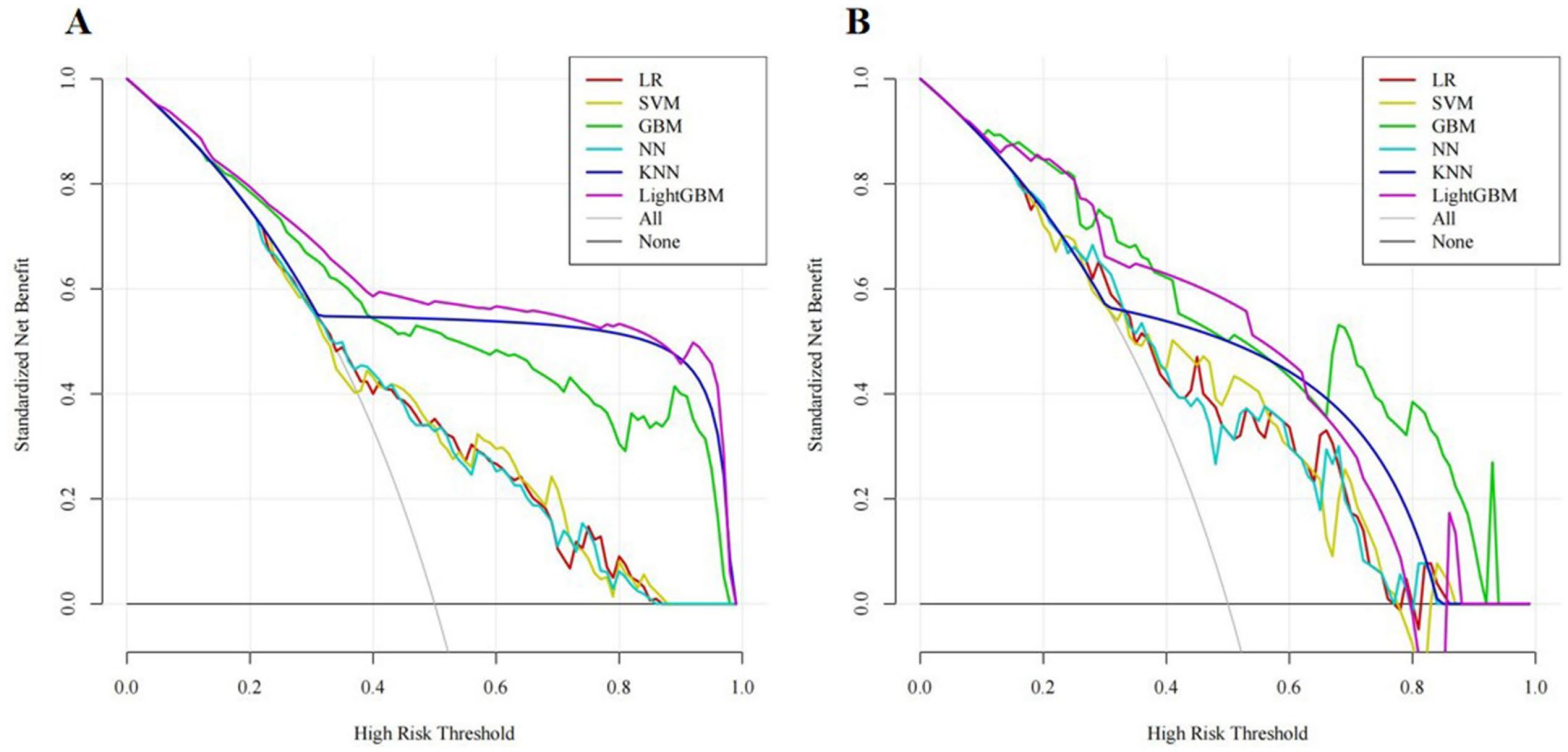
Decision curve analysis (DCA) for different machine learning models in the training set **(A)** and validation set **(B)**.

In addition, the clinical utility of each model was assessed through DCA. On the x-axis is the threshold probability, while the y-axis denotes the net benefit. In DCA, “all interventions” means that all patients receive interventions, while “no intervention” means that no patients receive interventions. Meanwhile, DCA demonstrated that our predictive models provided considerable net benefits across most threshold probabilities, suggesting their potential clinical utility. Among these models, GBM exhibited the highest net benefits in the validation set. As shown in Figure 5.

### Model interpretability

Assisted by explainable ML models, we utilized SHAP to analyse the GBM model, quantifying the contribution of each input variable to the model’s output. Furthermore, this interpretability framework offers two distinct forms of explanation: global explanations based on the feature level and local explanations for individual predictions.

**Figure 6:**
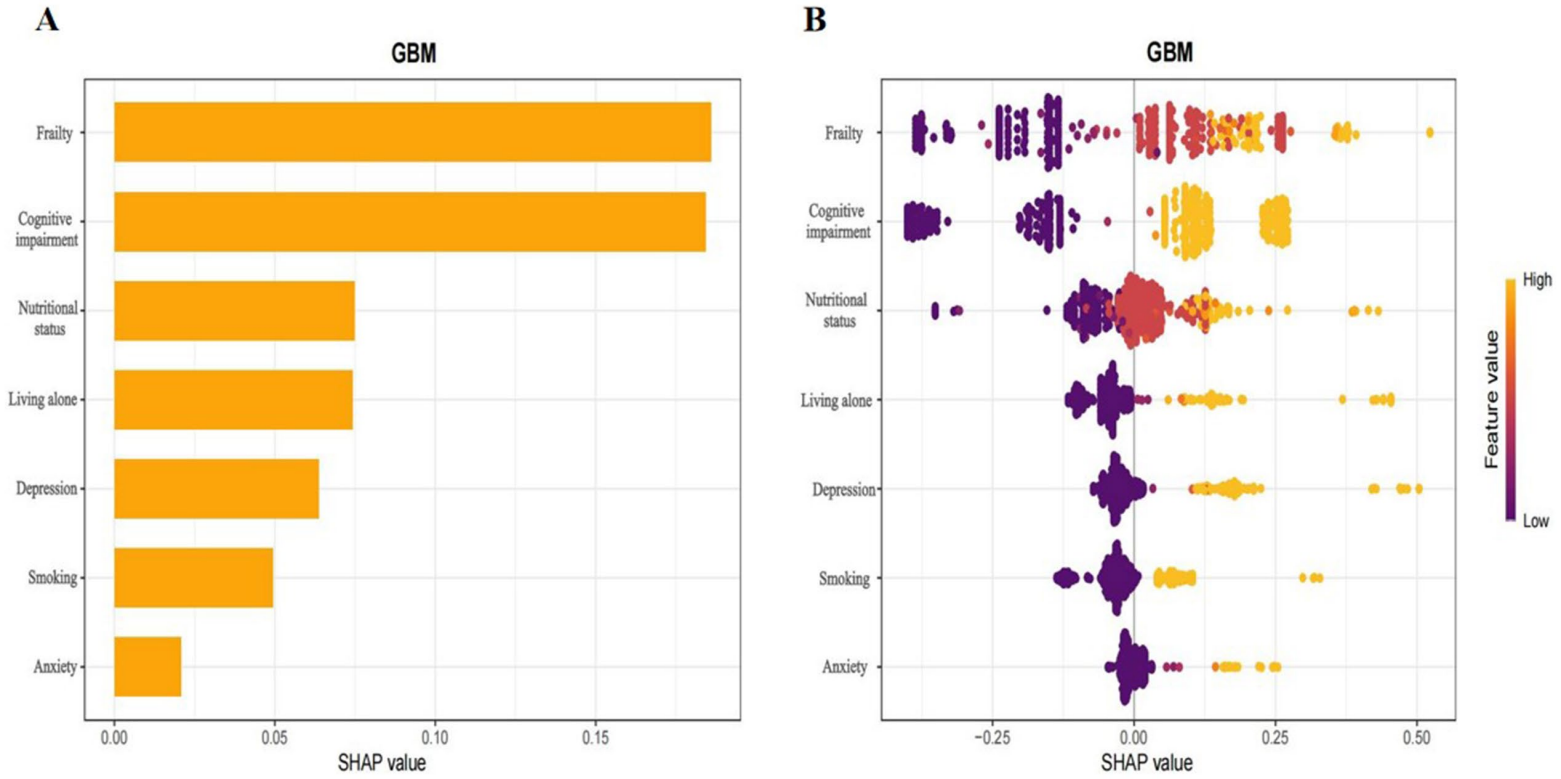
Interpretation of the GBM model based on SHAP.the x-axis denoted the contribution of each indicator to the prediction model. The y-axis indicated the characteristic value of each indicator, with all features presented. **(A)** Bar plot of feature importance, displaying the average SHAP values for each feature. **(B)** Summary plots showing the impact of each feature on model output. A positive value would augment the predicted result, whereas a negative value would reduce it. The orange dots represented high characteristic values, which indicated high risk, while the purple dots represented low characteristic values, which indicated low risk.

**Figure 7:**
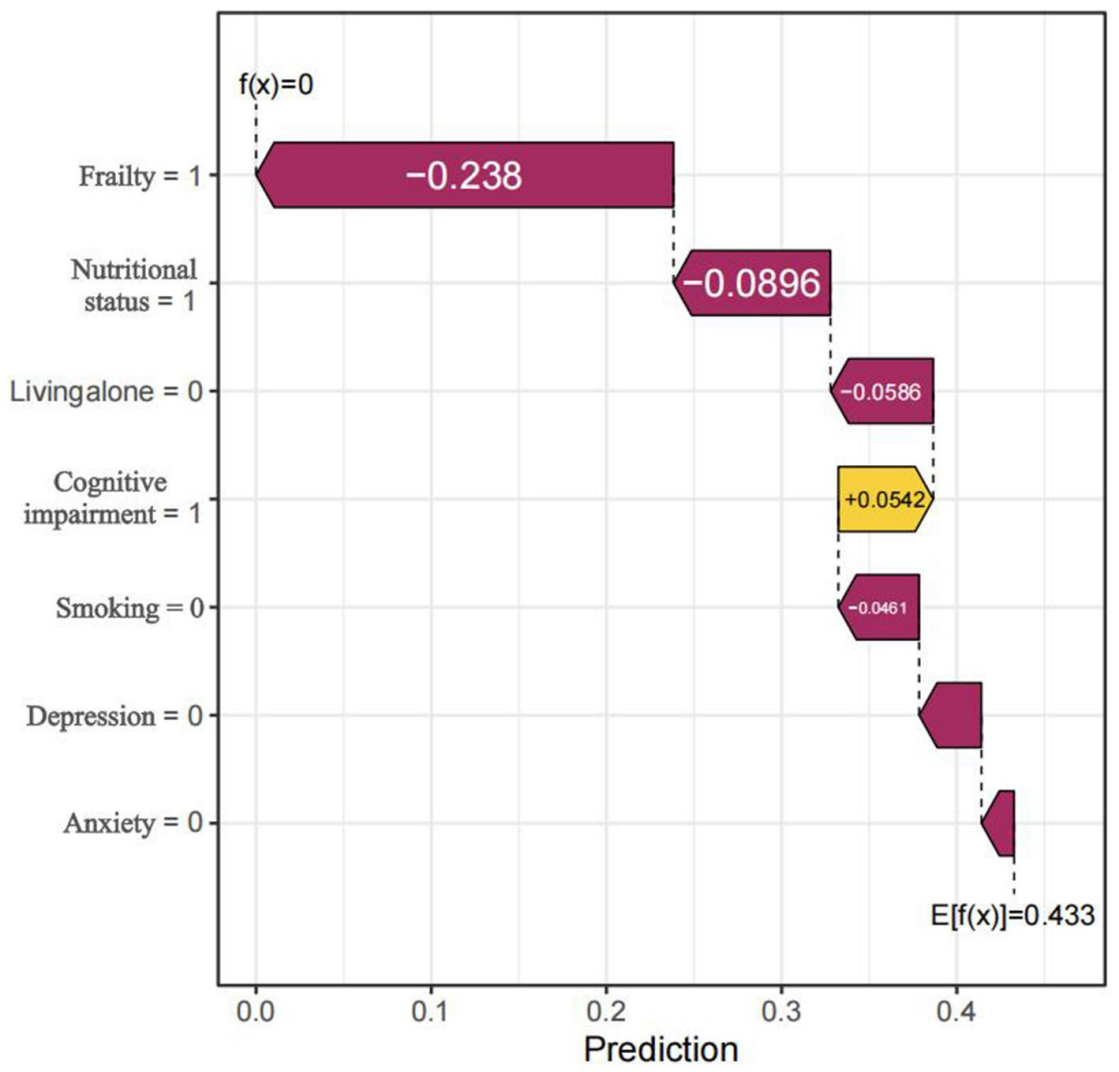
Local Prediction Explanation Plot, visualizing the SHAP waterfall plot for a single instance. The arrows indicated the influence of each feature on the prediction: orange arrows represented an elevated risk of the outcome, whereas purple arrows represented a reduced risk.

Global explanations of the model were depicted in Figure 6. Figure 6A highlighted the top seven factors influencing the model prediction. Figure 6B illustrated their corresponding effect values and interpretations. Within these figures. The findings indicated that the presence of frailty, cognitive impairment, poor nutrition, living alone, depression, smoking habits, and anxiety increased the risk of sleep disorders. Figure 7 was a local explanation SHAP waterfall plot at the individual level. Figure 7 provided an example to illustrate this point. The example featured an older adult individual with multimorbidity, no frailty, good nutritional status, living with family, cognitive impairment, a history of smoking, no depression, and no anxiety. The “no frailty” feature exerted a negative influence of −0.238 on the risk of sleep disorders, and the “good nutritional status” feature likewise exerted a negative influence of −0.238 on the risk of sleep disorders.

### Validation of predictors by logistic regression

To further validate the predictors, we performed an additional traditional logistic regression analysis on the included predictive factors. In the univariate analysis, living alone (OR = 3.32, 95%CI: 1.91–5.79, *p* < 0.001), smoking (OR = 1.90, 95%CI: 1.19–3.03, *p* = 0.007), depression (OR = 2.84, 95%CI: 1.68–4.80, *p* < 0.001), anxiety (OR = 5.46, 95%CI: 1.62–18.47, *p* = 0.006), cognitive impairment (OR = 2.66, 95%CI: 1.72–4.10, *p* < 0.001), pre-frailty (OR = 1.58, 95%CI: 1.01–2.48, *p* = 0.045), frailty (OR = 4.11, 95%CI: 2.22–7.61, *p* < 0.001), nutritional risk (OR = 1.62, 95%CI: 1.02–2.56, *p* = 0.04), and poor nutritional status (OR = 5.45, 95%CI: 2.63–11.29, *p* < 0.001) were identified as significant risk factors. However, in the multivariate analysis, only living alone (OR = 2.71, 95%CI: 1.49–4.94, *p* = 0.001), smoking (OR = 1.98, 95%CI: 1.20–3.27, *p* = 0.008), depression (OR = 2.03, 95%CI: 1.10–3.74, *p* = 0.024), and cognitive impairment (OR = 1.92, 95%CI: 1.20–3.07, *p* = 0.007) remained as independent risk factors (Supplementary Table S3).

## Discussion

This study represents the first application of ML methods to predict the risk of sleep disorders in Chinese older adult individuals with multimorbidity. We combined sociodemographic data, health behavior, mental health, and disease-related data and identified the seven most predictive features using LASSO and Boruta methods. These features include frailty, cognitive status, nutritional status, living alone, depression, smoking status, and anxiety. In comparing six ML algorithms (LR, SVM, GBM, NN, KNN, and LightGBM), we discovered that the GBM model exhibited superior predictive performance within the training dataset. Using the SHAP method, we assessed the model’s interpretability and determined the extent to which each predictor influenced the risk of sleep disorders. This provides a transparent explanation to support clinical decision-making.

The results of our study demonstrated that the prevalence of sleep disorders among older adults with multimorbidity was 28.7%. These findings exhibit some variations when compared with previous studies. A large multinational study involving 237,023 individuals with multimorbidity across 46 countries reported a higher prevalence of sleep disorders (43.9%) (57). Additionally, a cross-sectional study in China focusing on community-dwelling older adults with multimorbidity documented insomnia prevalence rates ranging from 32.22 to 52.71% (58). The observed inconsistencies in conclusions may be due to differences in the chronic conditions examined and the assessment methodologies employed. Notably, the sleep status of older adults with multimorbidity is generally poor, further supporting the strong association between multimorbidity and sleep disorders. The pathophysiological mechanisms of chronic diseases may directly disrupt sleep architecture, while polypharmacy can adversely affect sleep quality through various pathways (59, 60). Furthermore, psychosocial stressors commonly experienced by this population—including disease-related anxiety and social isolation—may exacerbate sleep disturbances via neuroendocrine mechanisms (61, 62). Importantly, our findings confirm the high prevalence of multimorbidity in the Chinese older adult population. Consequently, integrated management of multimorbidity warrants greater attention from both healthcare policymakers and practitioners in China.

There is a significant deficiency in predictive models for the early identification of sleep disorder risk among older adult individuals with multimorbidity. We developed ML predictive models to address this gap. The GBM model demonstrated superior performance, achieving an AUC of 0.881. Additionally, the model demonstrated good generalizability in the oldest age groups.

In comparison, the sleep disorder risk prediction model for coronary heart disease patients developed by Zheng et al. (20) using traditional logistic regression had an AUC of 0.851. Similarly, Šiarnik et al. (19) used traditional logistic regression to develop a sleep disorder risk model among stroke patients, with an AUC value of 0.810. These results highlight the advantages of machine learning (ML) models over conventional approaches, as traditional models often fail to account for the complex non-linear relationships between sleep disorders and their risk factors. GBM is particularly adept at managing intricate, large-scale datasets and can effectively identify both linear and non-linear associations (63), thereby enhancing the precision of sleep disorder predictions, as demonstrated in our study.

ML models possess the capability to surmount the limitations of conventional logistic regression models and offer precise risk estimations. However, they often fail to explain the source of risk. In our study, we addressed this issue by visualizing the risk estimates of the GBM model using SHAP values. We utilized SHAP bar plots and summary plots to identify the main factors contributing to the risk of sleep disorders in an older adult population with multimorbidity. Additionally, we employed waterfall plots to pinpoint the primary risk factors for individual patients. Consequently, this study stands as one of the most extensive applications of SHAP values thus far. To the best of our knowledge, although several previous studies have utilized SHAP values for model explanation, none have deployed SHAP plots to clarify both local and global interpretations. Recently, a study conducted across multiple hospitals in South Korea, focusing on predicting sleep disorders in hospitalized patients, utilized SHAP summary plots to depict the relationships between the top nine predictors and the overall outcome. However, this approach only permits a global interpretation of the risk of sleep disorders, thereby limiting its clinical applicability (64). Similarly, Troncoso-García et al. (65) established several ML models for sleep disorder prediction but did not incorporate global interpretation, which may restrict their clinical credibility and practicality. In contrast, our SHAP-based ML model not only offers a comprehensive and transparent explanation, aiding in the understanding of the key influencing factors of sleep disorders in older adult individuals with multimorbidity, but also accurately identifies the primary risk factors for individual patients. This provides a robust scientific basis for clinical decision-making and personalized interventions.

To further clarify the impact of predictive variables on the model, our study applied SHAP analysis to the best-performing GBM model. According to the feature importance ranking of the GBM model, we found that frailty, cognitive status, nutritional status, living alone, and depression were the five most significant predictors of sleep disorders. Frailty may lead to decreased sleep quality at night through chronic inflammation and decreased muscle function (66). In addition, patients with cognitive impairment often have circadian rhythm disorders, manifested as increased nighttime awakenings, fragmented sleep, and reduced REM sleep, which may be related to neuroinflammation caused by amyloid beta deposition (67). The nutritional status of an individual can significantly influence their sleep quality and duration. Zhao et al. (68) that nutrition can significantly influence hormone levels and inflammation status, both of which can contribute directly or indirectly to the development of insomnia. Living alone may exacerbate insomnia symptoms through social isolation and psychological stress. A large-scale cohort study showed that the probability of older adult people living alone reporting insomnia symptoms was significantly higher than that of those living with others (69). Our research indicated that depression is a significant factor in the development of sleep disorders among the older adult. Studies have found that the hypothalamus-p. Thetary-adrenal (HPA) axis function is abnormal in depressed patients, leading to increased cortisol levels, which in turn affect circadian rhythms and sleep structure (70). Therefore, individuals with mental health problems should pay attention to sleep issues. This study further verified the importance of these factors to model predictions through SHAP analysis, suggesting that in the sleep health management of older adult patients with multimorbidity, attention should be paid to mental health, lifestyle, and social support factors to optimize intervention strategies. The logistic regression analysis further confirmed that living alone, smoking, depression, poor nutritional status, anxiety, frailty, and cognitive impairment are risk factors for sleep disorders, indicating good consistency between the machine learning model constructed in this study and traditional analytical methods. Moreover, careful consideration of these factors could contribute to the prevention of sleep disorders. For instance, avoiding living alone, quitting smoking, preventing depression, and preventing cognitive impairment can all help prevent sleep disorders.

Accurately predicting modifiable risk factors for sleep disorders in older adults with multimorbidity is crucial. Our model effectively identifies high-risk individuals, offering significant benefits for this population. Moreover, healthcare professionals often struggle to comprehend how machine learning models generate predictions based on their internal structures. In contrast, our approach, which relies on SHAP, is simple and highly interpretable, making it more suitable for routine clinical use. For instance, the SHAP global interpretability method can help clinicians identify common risk factors within the patient population, guiding preventive measures and public health interventions. Meanwhile, the SHAP local interpretability method enables clinicians to tailor their approach to individual patients, ensuring that interventions are more precise and effective. Healthcare professionals can assess a patient’s likelihood of developing sleep disorders by examining the proportion of SHAP values attributed to different predictors. When older adults with multimorbidity begin to exhibit frailty, cognitive impairment, poor nutritional status, living alone, or experience depression—whether individually or in combination—healthcare professionals should be alerted and take proactive measures to prevent sleep disorders. Previous research has also confirmed the effectiveness of interventions targeting these predictors. Rezaei-Shahsavarloo et al. (71) demonstrated in a systematic review that multidimensional interventions significantly improved physical function and reduced frailty in hospitalized older adults, potentially enhancing sleep quality indirectly. Halson (72) reviewed the effects of nutritional interventions on sleep quality and quantity in athletes, indicating that carbohydrate, tryptophan, and melatonin may improve sleep onset and quality. O’Caoimh et al. (73) conducted a systematic review of non-pharmacological interventions for sleep disturbances in individuals with mild cognitive impairment and dementia, finding that multimodal approaches, particularly those incorporating light therapy, significantly improved sleep quality. Finally, healthcare professionals should use our model judiciously. It should serve as an auxiliary tool rather than the sole basis for decision-making. Over-reliance on model predictions may overlook individual patient differences, such as variables not included in the model. For example, while living alone was identified as a risk factor, the model cannot distinguish between voluntary solitude and involuntary loneliness. Healthcare professionals need to combine humanistic care with personalized assessments. Potential misinterpretation by non-expert users should also be avoided.

## Limitations

This study has some limitations. Firstly, the cross-sectional design utilized does not allow for definitive conclusions regarding causality. Future longitudinal research is necessary to delve deeper into our findings. Secondly, the representativeness of the sample is somewhat constrained. Given that the study population was exclusively composed of Chinese older adult individuals, the model’s cultural applicability may be somewhat limited. Therefore, we recommend that future studies conduct multicenter collaborative studies in populations with diverse cultural and ethnic backgrounds to validate the model. Finally, this study is the lack of external validation of the predictive model. While the model demonstrated robust performance on the internal validation set, its generalizability to other populations or settings remains uncertain. External validation using independent datasets from different regions, healthcare systems, or demographic groups is essential to confirm the model’s reliability and applicability in diverse clinical environments. This limitation should be addressed in future research to ensure the model’s broader clinical utility.

## Conclusion

This study identified several significant risk factors for sleep disorders in older adult individuals with multimorbidity, including frailty, cognitive status, nutritional status, living alone, depression, smoking status, and anxiety. We implemented ML approaches to predict sleep disorders risk in this population, evaluating various algorithms including LR, SVM, GBM, NN, KNN, and LightGBM. The GBM model demonstrated exceptional performance, achieving an AUC of 0.881 and an accuracy of 0.798 in predicting sleep disorder risk. Furthermore, the study employed SHAP techniques to enhance model interpretability. This method provided both global and local explanations of the model’s decision-making processes, thereby improving the transparency and clinical utility of the predictive system.

## Data Availability

The original contributions presented in the study are included in the article/Supplementary material, further inquiries can be directed to the corresponding author.
