# Correction: Development and validation of an explainable machine learning model for predicting the risk of sleep disorders in older adults with multimorbidity: a cross-sectional study

**DOI:** 10.3389/fpubh.2025.1684776

**Published:** 2025-08-29

**Authors:** Xia Wang, Dan Zhang, Liu Lu, Shujie Meng, Yong Li, Rong Zhang, Jingjie Zhou, Qian Yu, Li Zeng, Jiang Zhao, Yu Zeng, Ru Gao

**Affiliations:** ^1^School of Basic Medical Sciences and School of Nursing, Chengdu University, Chengdu, China; ^2^Rehabilitation Department, Sichuan Provincial People's Hospital East Sichuan Hospital and Dazhou First People's Hospital, Dazhou, China; ^3^Nursing Department, The Fourth People's Hospital of Yibin, Yibin, China; ^4^Rehabilitation College, Sichuan Health Rehabilitation Vocational College, Zigong, China; ^5^Tellyes Scientific Inc., Tianjin, China; ^6^Nursing Department, The People's Hospital of Wenjiang Chengdu, Chengdu, China

**Keywords:** machine learning, multimorbidity, older adults, sleep disorder, prediction model

In the published article, there was an error in the affiliation for author Dan Zhang. Instead of “Nursing Department, The People's Hospital of Wenjiang Chengdu, Chengdu, China”, it should be “Rehabilitation Department, Sichuan Provincial People's Hospital East Sichuan Hospital and Dazhou First People's Hospital, Dazhou, China”.

The original article has been updated.

