# Supplementary material for: Development and validation of an explainable machine learning model for predicting the risk of sleep disorders in older adults with multimorbidity: a cross-sectional study
Source: Front Public Health. 2025 Aug 11;13:1619406. doi: 10.3389/fpubh.2025.1619406 (PMC12375460; doi:10.3389/fpubh.2025.1619406)
Supplement: Supplementary file 1 [file Data_Sheet_1.doc]

Supplementary Material

**Development and validation of an explainable machine learning model for predicting the risk of sleep disorders in older adults with multimorbidity: a cross-sectional study**

# 1 Supplementary Tables

**Supplementary Table 1.** Comparison of characteristics between sleep disorders and non-sleep disorders groups in the training set following SMOTE-based oversampling for data balance.

| Variables | Non-sleep disorders  (n=184) | Sleep disorders  (n=184) | *p* |
| --- | --- | --- | --- |
| Living alone (%) |  |  | <0.001 |
| No | 7 (3.8) | 54 (29.3) |  |
| Yes | 177 (96.2) | 130(70.7) |  |
| Smoking (%) |  |  | <0.001 |
| No | 151(82.1) | 117(63.6) |  |
| Yes | 33 (17.9) | 67 (36.4) |  |
| Depression (%) |  |  | <0.001 |
| No | 161(87.5) | 121(65.8) |  |
| Yes | 23 (12.5) | 63 (34.2) |  |
| Anxiety (%) |  |  | <0.001 |
| No | 181(98.4) | 160(87.0) |  |
| Yes | 3 (1.6) | 24 (13.0) |  |
| Cognitive impairment (%) |  |  | 0.002 |
| No | 95(51.6) | 64(34.8) |  |
| Yes | 89 (48.4) | 120 (65.2) |  |
| Frailty (%) |  |  | <0.001 |
| No | 98 (53.3) | 67 (36.5) |  |
| Pro | 67 (36.4) | 70 (38.0) |  |
| Yes | 19 (10.3) | 47 (25.5) |  |
| Nutritional status (%) |  |  | 0.001 |
| Good | 69 (37.5) | 47 (25.5) |  |
| Risk | 98 (53.3) | 97 (52.7) |  |
| Bad | 17 (9.2) | 40 (21.5) |  |

**Supplementary Table 2. DeLong test for comparing AUCs of ROC curves among six machine learning models.**

| Comparisons | *Z* score | *p* value |
| --- | --- | --- |
| GBM model vs. LightGBM model | -2.168 | 0.030 |
| GBM model vs. KNN model | 3.452 | <0.001 |
| GBM model vs. NN model | 6.083 | <0.001 |
| GBM model vs. LR model | 6.195 | <0.001 |
| GBM model vs. SVM model | 6.053 | <0.001 |

GBM, gradient boosting machine; LghtGBM, light gradient boosting machine; KNN, k-Nearest Neighbors; NN, neural network; LR, logistic regression; SVM, support vector machine; AUC, the area under the curve.

**Supplementary Table 3. **Logistic regression analysis of risk factors for sleep disorders in older adults with multimorbidity.****

| Variables | Univariate analysis OR (95%CI) | *P* | Multivariate analysis OR (95%CI) | *P* |
| --- | --- | --- | --- | --- |
| Living alone |  |  |  |  |
| No | Reference |  |  |  |
| Yes | 3.32 (1.91-5.79) | <0.001 | 2.71 (1.49-4.94) | 0.001 |
| Smoking |  |  |  |  |
| No | Reference |  |  |  |
| Yes | 1.90 (1.19-3.03) | 0.007 | 1.98 (1.20-3.27) | 0.008 |
| Depression |  |  |  |  |
| No | Reference |  |  |  |
| Yes | 2.84 (1.68-4.80) | <0.001 | 2.03 (1.10-3.74) | 0.024 |
| Anxiety |  |  |  |  |
| No | Reference |  |  |  |
| Yes | 5.46 (1.62-18.47) | 0.006 | 1.81 (0.46-7.19) | 0.4 |
| Cognitive impairment |  |  |  |  |
| No | Reference |  |  |  |
| Yes | 2.66 (1.72-4.10) | <0.001 | 1.92 (1.20-3.07) | 0.007 |
| Frailty |  |  |  |  |
| No | Reference |  |  |  |
| Pre | 1.58 (1.01-2.48) | 0.045 | 1.10 (0.67-1.81) | 0.706 |
| Yes | 4.11 (2.22-7.61) | <0.001 | 2.02 (0.97-4.20) | 0.061 |
| Nutritional status |  |  |  |  |
| Good | Reference |  |  |  |
| Risk | 1.62 (1.02-2.56) | 0.04 | 1.19 (0.72-1.95) | 0.501 |
| Bad | 5.45 (2.63-11.29) | <0.001 | 2.20 (0.95-5.07) | 0.064 |

BMI, body mass index; OR, odds ratio; CI, confidence interval.

# 2 Supplementary Figures

#
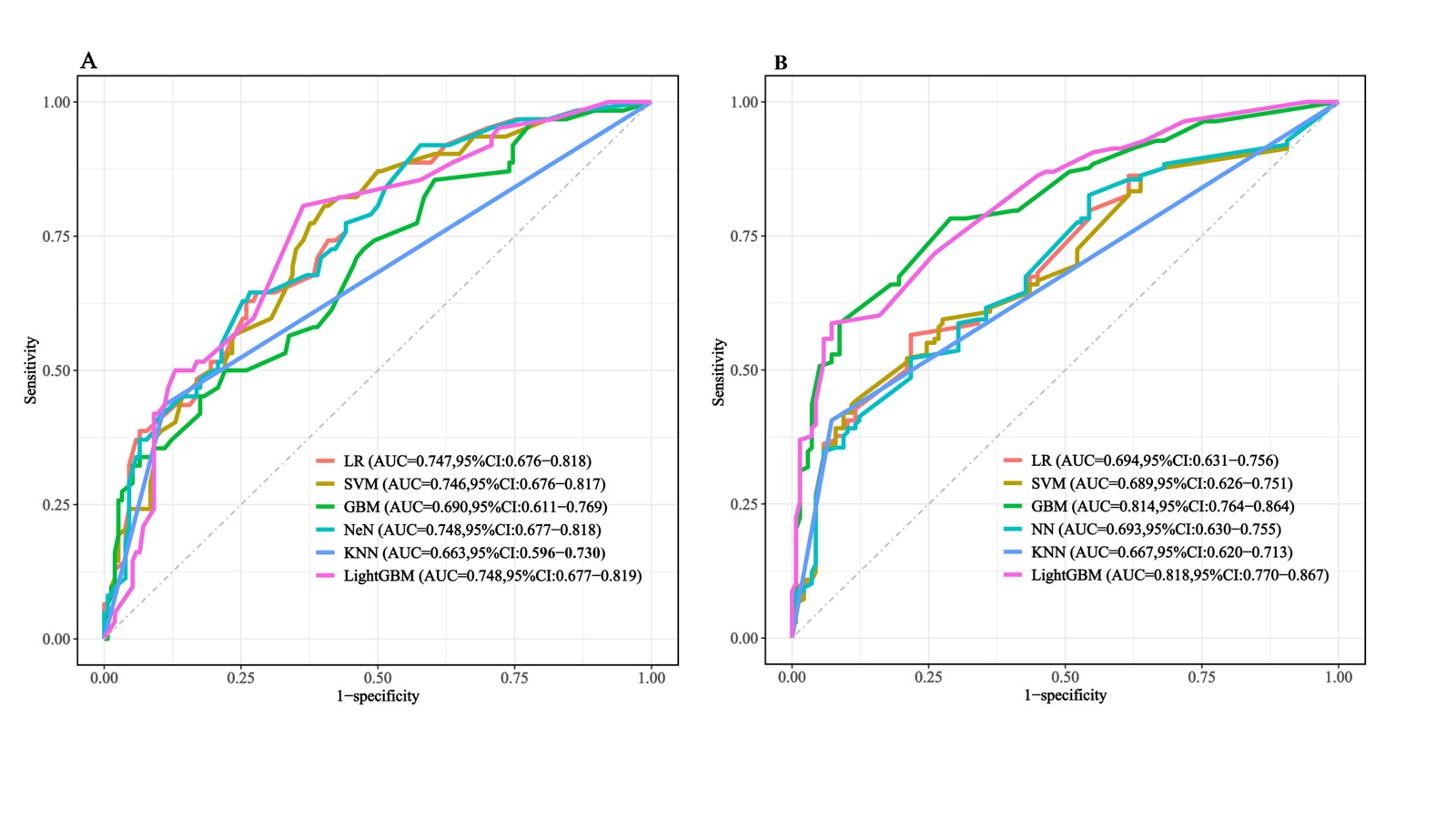


**Supplementary Figure 1.** Receiver-operating characteristic (ROC) curves of machine learning models validated in sex subgroups. (A) Female; (B) Male.


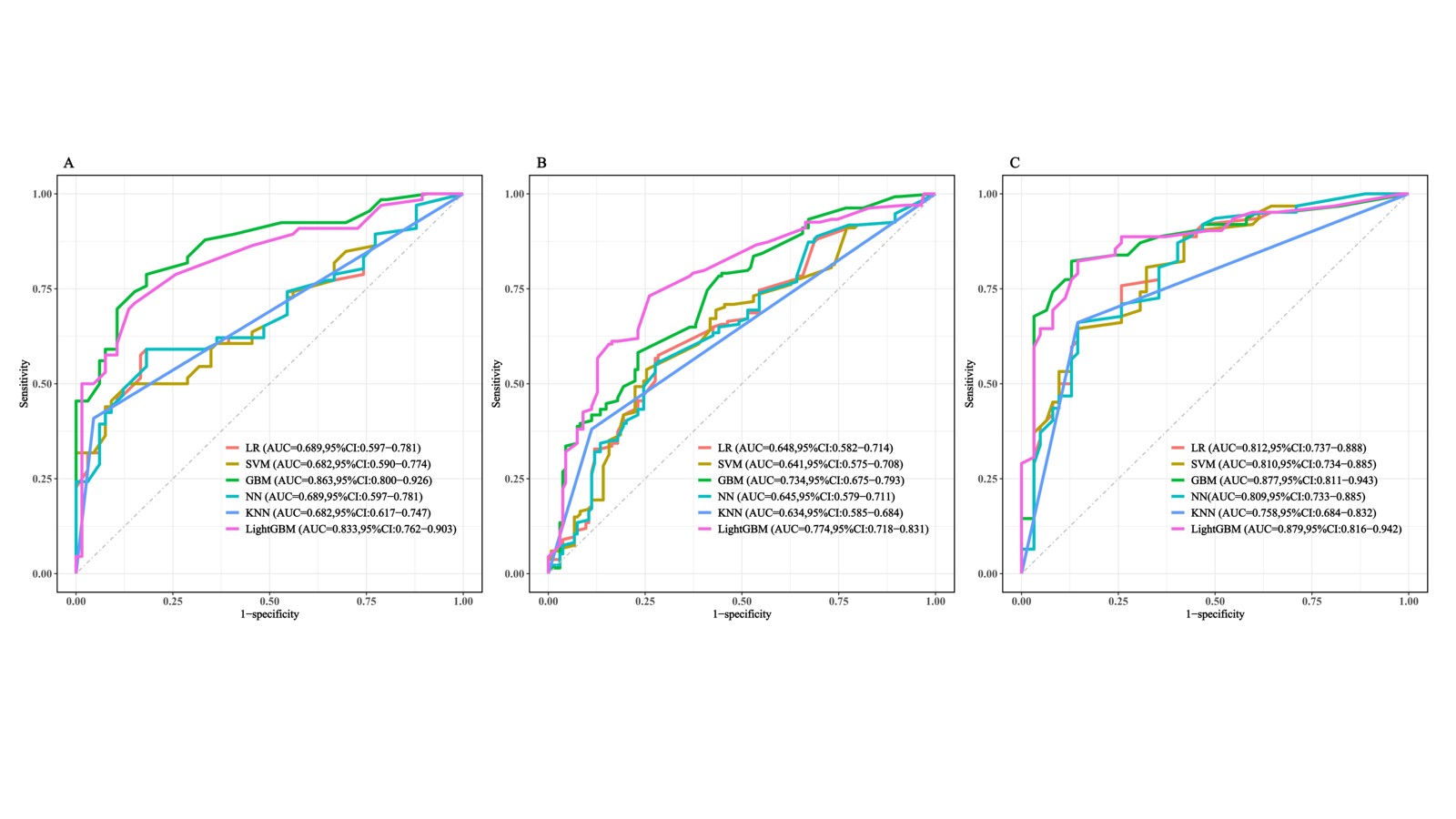


**Supplementary Figure 2.** Receiver-operating characteristic (ROC) curves of machine learning models validated in age subgroups. (A) 60-69 years; (B) 70-79 years; (C) ≥80 years.
